# Tobacco Control: a finite project with the end on the horizon

**DOI:** 10.1136/tc-2023-058514

**Published:** 2023-12-12

**Authors:** Marita Hefler

**Affiliations:** Wellbeing and Preventable Chronic Diseases Division, Menzies School of Health Research, Charles Darwin University, Casuarina, NT 0810, Australia

**Keywords:** Addiction, Advertising and Promotion, Advocacy

Assuming editorship of any journal carries the responsibility of continuing to advance the field in new directions, building on the foundations laid by predecessors. This is particularly so for *Tobacco Control,* as the leading specialist journal at a pivotal time in the history of the tobacco epidemic. It is my hope and ambition as *Tobacco Control’s* fourth editor-in-chief that as the journal enters its 32nd year, it has already passed the halfway mark of its life.

In 2009, the first issue edited by my predecessor Professor Ruth Malone presented two opposing perspectives. One argued for phasing out of cigarette sales^1^; the other that ‘prohibition’ would be counterproductive and incremental regulatory measures are more politically feasible and effective.^2^ At the time, it was more a theoretical thought experiment than a debate with immediate policy impact. However, it set the scene for Tobacco Control’s direction in the years that followed. While the regulatory incrementalism advocated in the second perspective has continued to dominate tobacco control policy globally, today the balance is shifting in favour of the former perspective, with several jurisdictions implementing or planning staged phase-outs of tobacco product sales.^3^ Tobacco industry framing of ending sales of the most lethal consumer product in history as a path to catastrophic ‘prohibition’ has been thoroughly rebutted.^4^ Professor Malone is a thought leader, and her 14 years at the helm of this journal saw much scholarship published that stimulated thinking, generated evidence and advanced moves towards the tobacco endgame. The field owes an extraordinary debt to her visionary stewardship. Stepping into her shoes is personally daunting, somewhat lessened by having been inspired and learned from her generous mentorship over many years. Thankfully, *Tobacco Control* will continue to benefit from her wisdom as new Editor Emeritus, joining her predecessor Professor Simon Chapman.

There are many reasons for optimism that the endpoint for the tobacco epidemic is within sight. Once groundbreaking policies begin to be adopted by multiple jurisdictions, they typically rapidly become normalised.^5^ Sometimes it takes many years to achieve critical mass, as in the case of pictorial health warning labels on tobacco packs. In others, such as plain packaging, uptake by others occurs relatively quickly.^5^ Small jurisdictions can inspire widespread change, when once ‘impossible’ policies are shown to be both possible and supported by robust evidence. In my part of the world, Aotearoa New Zealand introduced a nationwide smoke-free prisons policy in 2011. It was followed by Australia’s least populated jurisdiction the Northern Territory in 2013, with other Australian states and territories rapidly following suit. Today, smoke-free correctional facilities are the norm in both countries.

The first national tobacco ‘endgame’ policy was Bhutan’s 2004 ban on tobacco sales. It failed to reduce high tobacco use, and for many years was a global policy anomaly before being suspended in 2020 as part of COVID-19 emergency measures.^6^ Although Bhutan’s policy approach has not been adopted elsewhere, it likely provides valuable and largely unexplored policy lessons, and is now one of many examples in an expanding global ‘living policy laboratory’ of tobacco endgame approaches being implemented at local, state and national levels. *Tobacco Control* welcomes research that draws lessons from these innovative, paradigm-shifting approaches and presents evidence and ideas for new policy directions. This will continue building the evidence base for the WHO Framework Convention on Tobacco Control (FCTC), reflecting the shared history of the journal and the treaty.^7^ Endgame measures align with a new focus on FCTC Article 2.1, which is formally on the agenda for the 10th Conference of the Parties—the first time since the original treaty negotiations it has been included.

There are numerous pathways to achieving the tobacco endgame. The challenges are much greater for countries with limited resources, high levels of tobacco industry interference and high tobacco use prevalence—but there is increasing recognition that setting targets and plans is essential, whatever the current context.^8^ Many low-income and middle-income countries offer examples of tobacco control policy innovation and leadership.^5 9^ *Tobacco Control* has a strong history of highlighting such initiatives and supporting less experienced authors, including those in resource-constrained settings, to publish in the journal. The News Analysis section and blog have both served as entry points for publication. Similarly, the Industry Watch and Advocacy in Action sections offer opportunities for those working outside of research, particularly in civil society organisations, to showcase innovation to a global audience, share examples of research translation and contribute to awareness and momentum for different policy approaches. Our commitment to supporting authors to publish through these avenues—with guidance as needed—will continue.

Globally, there are increasing calls for decolonisation of global health; that is, to see the end of supremacist remnants of colonisation in favour of a global health architecture underpinned by justice, equity, diversity and inclusion, both within and between countries.^10^ This means conceptualising expertise as an equal exchange of knowledge between high-income and low-income countries, and a shared imperative to meet the needs of people experiencing disadvantage, regardless of setting. In practical terms, it also means addressing the frequent imbalance in authorship that occurs between research partners in high-income and low-income countries, or even the complete absence of authors from low-income countries where research was undertaken. We will be introducing new publishing policies to address these issues. Work has already begun with publications in 2023 about ethical publishing in Indigenous contexts^11^ and decentring whiteness in tobacco control.^12^ Together with a new policy of person-first language,^13^ these initiatives aim to contribute to ensuring equity and minimising the gap between researchers and the people served by tobacco control policy.

Continuing to be the journal of choice for high-quality research exploring the impact of tobacco use on health, the economy, environment and society—as well as the changing nature of the tobacco industry and its products—also means recognising and responding to increasing challenges within academia in both high-income and low-income settings. It is crucial that the pipeline of emerging researchers is well supported to generate the evidence needed to see the tobacco epidemic enter the history books. In 2024, I will be consulting with the *Tobacco Control* editorial board about introducing initiatives which offer mentoring and other opportunities to build authorship capacity of less experienced researchers. We will also develop initiatives for new reviewers that offer learning opportunities, career development support and formal recognition. Expanding the pool of quality reviewers is crucial, given the increasing difficulty of obtaining sufficient reviewers for papers—a problem we share with other journals in the field.^14^ On that note, a plea to experienced and senior authors, particularly those whose research is prolific in *Tobacco Control*: please be good academic citizens and accept review invitations for a similar number of papers to those you publish!

Of course, the biggest challenge in our field continues to be the tobacco industry and its direct and indirect strategies to distort evidence and interfere in public health policy. *Tobacco Control* will always have space for different interpretations of evidence, particularly in relation to the perennially contentious notion of ‘harm reduction’. However, good faith debates are undermined by those in tobacco control willing to go through the revolving door to positions with the tobacco industry and organisations funded by it. Despite its protestations that it is changing and part of the solution to a ‘smoke-free future’, the tobacco industry’s most lethal product continues to dominate sales, in the context of a focus on market growth for all its products. Whatever one’s view of the continually evolving range of heated tobacco products, e-cigarettes and other non-combusted products, the nicotine product market is mostly one of degrees of harm rather than no harm. Ending the tobacco epidemic means reducing overall demand for nicotine, in much the same way as tackling climate change requires the world to move away from a consumption-centred, growth-at-all-costs mindset.^15^ Tobacco industry allies and sympathisers may legitimately argue that no company can voluntarily decide to stop sales of its most dangerous products, or abandon customer recruitment for new products, without abrogating its responsibility to shareholders. That is precisely the point. The industry’s responsibility is not to the greater good, public health, governments or even its customers. It should not be controversial to rigorously maintain the firewall between the tobacco industry and legitimate science and evidence generation for effective public health policy-making.
